# Facial restoration of morphea and Parry-Romberg syndrome with resilient hyaluronic acid: a case report

**DOI:** 10.1097/JW9.0000000000000279

**Published:** 2026-06-26

**Authors:** Eryn Patin, Kathyana P. Santiago Mangual, Arianne Shadi Kourosh, Young H. Lim, Sandy Tsao

**Affiliations:** a Department of Dermatology, Massachusetts General Hospital, Boston, Massachusetts; b The University of Texas Southwestern Medical School, Dallas, Texas; c Department of Dermatology, Beth Israel Deaconess Medical Center, Boston, Massachusetts; d Department of Medicine, Yale University School of Medicine, New Haven, Connecticut; e Harvard Medical School, Boston, Massachusetts; f Harvard T.H. Chan School of Public Health, Boston, Massachusetts

**Keywords:** hyaluronic acid, immunogenicity, Morphea, Parry-Romberg syndrome, scleroderma

**What is known about this subject in regard to women and their families?**

- Morphea, a rare chronic inflammatory immune-mediated connective tissue disorder affecting the dermis and subcutaneous tissues, can co-occur with Parry-Romberg syndrome (PRS), which is characterized by progressive hemifacial atrophy involving underlying bone and cartilage. Notably, both conditions demonstrate a higher prevalence among women.

**What is new from this article as messages for women and their families?**

- Injectable hyaluronic acid dermal fillers offer a minimally invasive and effective approach for addressing soft tissue atrophy and volume loss in morphea and PRS, though efficacy may be influenced by the selected filler’s rheological characteristics and immunogenic profile.
- To our knowledge, this report is the first to describe the use of resilient hyaluronic acid, a formulation with reduced proinflammatory and immunogenic potential, for aesthetic correction in patients with morphea and PRS.

## Introduction

Morphea, a chronic inflammatory immune-mediated connective tissue disease affecting the dermis and subcutaneous tissues, can occur alongside Parry-Romberg syndrome (PRS), manifesting with hemifacial atrophy extending to the bone and cartilage.^1,2^ Although immunomodulatory treatments may halt disease progression, lesions often remain with dyspigmentation and disfigurement, adversely affecting patients’ quality of life.^1^ Treatment options for facial symmetry restoration include autologous fat and bone grafting, which are invasive and costly.^1^ Injectable hyaluronic acid (HA) dermal fillers provide an effective and minimally invasive alternative, though data regarding long-term safety and durability in immune-mediated disease remain limited.^3^ This report highlights a case of facial restoration in a patient with stabilized co-occurring morphea and PRS with the use of resilient hyaluronic acid (RHA2 and RHA3; Teoxane SA, Geneva, Switzerland).

## Case

**Fig. 1.**
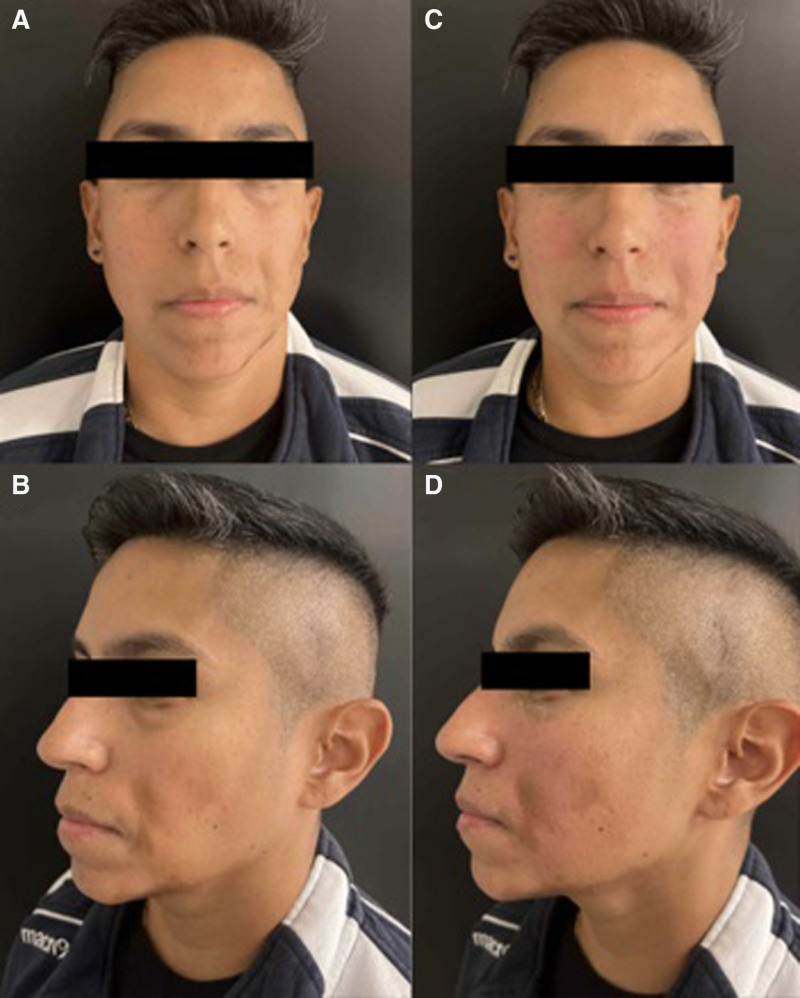
Before (A, B) and after (C, D) resilient hyaluronic acid (RHA) injections for correction of morphea and Parry-Romberg syndrome (PRS) affecting the left inferior eyelid, mid-lower cheek, and nasolabial fold.

A 38-year-old woman with stable morphea and PRS, affecting her left inferior eyelid, nasolabial fold, and chin, sought restorative treatment for atrophy and volume loss. After achieving disease stability with methotrexate therapy for a year, with no evidence of active inflammation or disease progression, she expressed interest in minimally invasive procedures. Physical examination revealed hemifacial atrophic plaques without erythema and with preserved overall symmetry of underlying bone structures (Fig. 1A and B).

After obtaining informed consent, 0.4 mL of RHA2 was injected to the left inferior eyelid and 1.8 mL of the more viscous RHA3 to her left mid and lower cheeks and nasolabial fold. For additional symmetry, 0.2 mL of RHA2 was injected to the right inferior eyelid (Fig. 1C and D). The patient reported great satisfaction with results immediately post-treatment, and maintenance of clinical improvement was observed at 6-month follow-up with no evidence of disease recurrence.

## Discussion

HA indirectly stimulates connective tissue formation and can be used for cosmetic restoration in patients with morphea and PRS, but should only be administered when inflammation is quiescent to avoid triggering disease reactivation.^1^ Compared with other HA fillers, RHA contains the lowest concentration of low-molecular-weight (LMW) HA, a proinflammatory molecule capable of triggering an immune response.^3,4^ RHA is designed to preserve high-molecular-weight (HMW) HA chains, forming an entangled network of elongated polymers that more closely resembles the natural conformation of HA.^5^ This network requires fewer 1,4-butanediol diglycidyl ether crosslinkers, thus decreasing gel stiffness, enabling dynamic sliding, and preventing degradation of HMW HA chains.^5^ The higher relative content of HMW HA, which more closely resembles the natural conformation of HA and is known to inhibit proinflammatory mediators, may also enhance tissue biointegration and reduce immunogenicity,^3,5^ vital considerations for patients with immune-mediated disease. Furthermore, RHA exhibits greater gel cohesivity, which may reduce susceptibility to degradation and subsequent exposure to proinflammatory byproducts capable of triggering adverse immune reactions, potentially supporting longer-lasting, natural-appearing, and safer results.^3,4^ Importantly, these properties are inferred from prior rheologic and immunogenicity studies rather than directly demonstrated in this single case. Given the permanent nature of PRS, result longevity, alongside immunogenicity, may be an important consideration when selecting filler materials to minimize the frequency of filler treatments.

Importantly, disease stability was maintained throughout the 6-month follow-up period, with no clinical evidence of morphea or PRS reactivation, supporting the short-term tolerability of RHA in this patient. Nevertheless, this report is limited by its single-patient design, absence of objective outcome measures, and relatively short follow-up duration. As such, conclusions regarding long-term safety, durability, and immunogenicity should be interpreted cautiously.

Selecting the most appropriate filler requires a thorough understanding of the disease pathophysiology, associated changes in facial anatomy, and the filler’s physical and rheological properties. To our knowledge, this report is the first to describe the use of RHA for the corrective treatment of morphea and PRS, offering a safe, cost-effective, and durable solution for restoring facial symmetry and expressiveness.

## Conflicts of interest

None.

## Funding

None.

## Study approval

The author(s) confirm that any aspect of the work covered in this manuscript that has involved human patients has been conducted with the ethical approval of all relevant bodies.

## Author contributions

All authors participated in the performance of research, data analysis and the writing of the manuscript.

## Patient consent

Consent for the publication of recognizable patient photographs or other identifiable material was obtained by the authors with the understanding that this information may be publicly available.
